# Supplementary material for: Impact of Atorvastatin on Skeletal Muscle Mitochondrial Activity, Locomotion and Axonal Excitability—Evidence from ApoE-/- Mice
Source: Int J Mol Sci. 2022 May 12;23(10):5415. doi: 10.3390/ijms23105415 (PMC9141374; doi:10.3390/ijms23105415)
Supplement: Supplementary file 1 [file ijms-23-05415-s001.zip › ijms-1701870-supplementary.pdf]

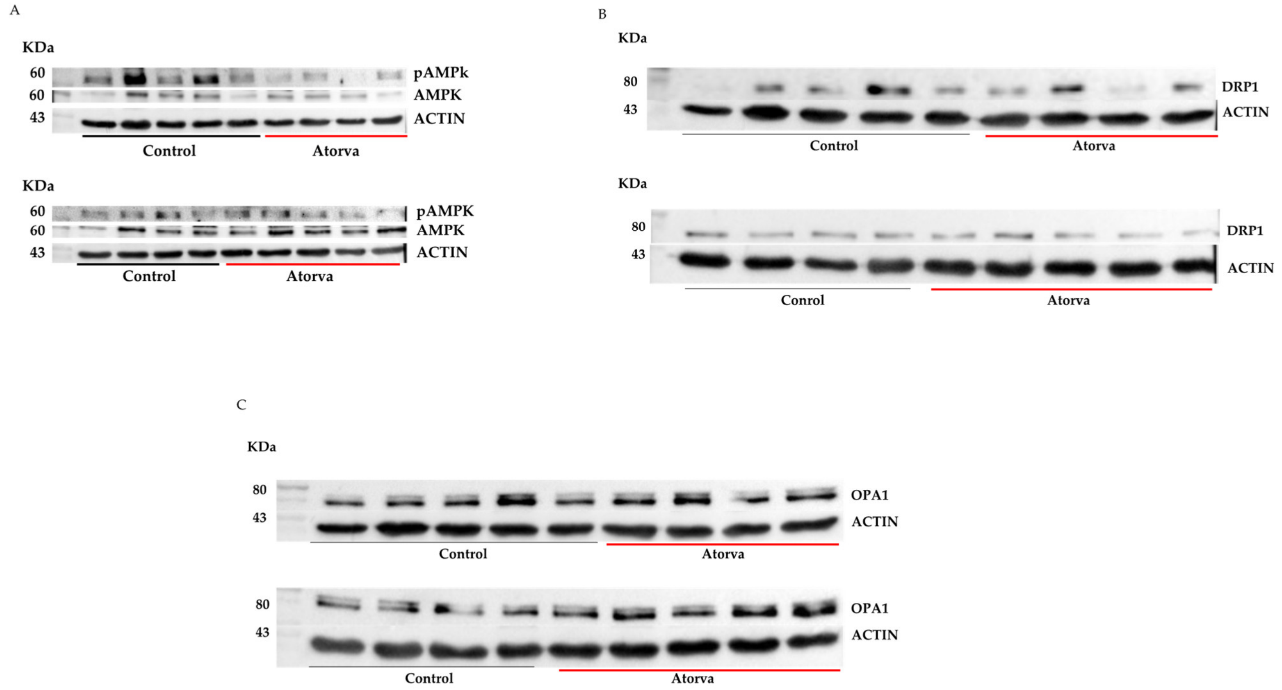

Figure S1. Western blot analyses of pAMPK/AMPK, DRP-1 and OPA-1. Protein expression of pAMPK/AMPK (**A**), DRP-1 (**B**) and OPA1 (**C**) in quadricepts of *ApoE*$^{-/-}$ mice fed HFHC and HFHC plus atorvastatin. Actin was used as a housekeeping. Proteins were extracted from 20 mg of tissue and Western blots analyses have been performed on 9 quadriceps in each group. AMPK, serine/threonine kinase AMP-activated protein kinase; DRP-1, dynamin related protein 1; HFHC, high-fat high cholesterol; OPA1, Optic atrophy 1. kDa, kilodalton; Control, high-fat high cholesterol diet; Atorva, high-fat high cholesterol diet plus atorvastatin.

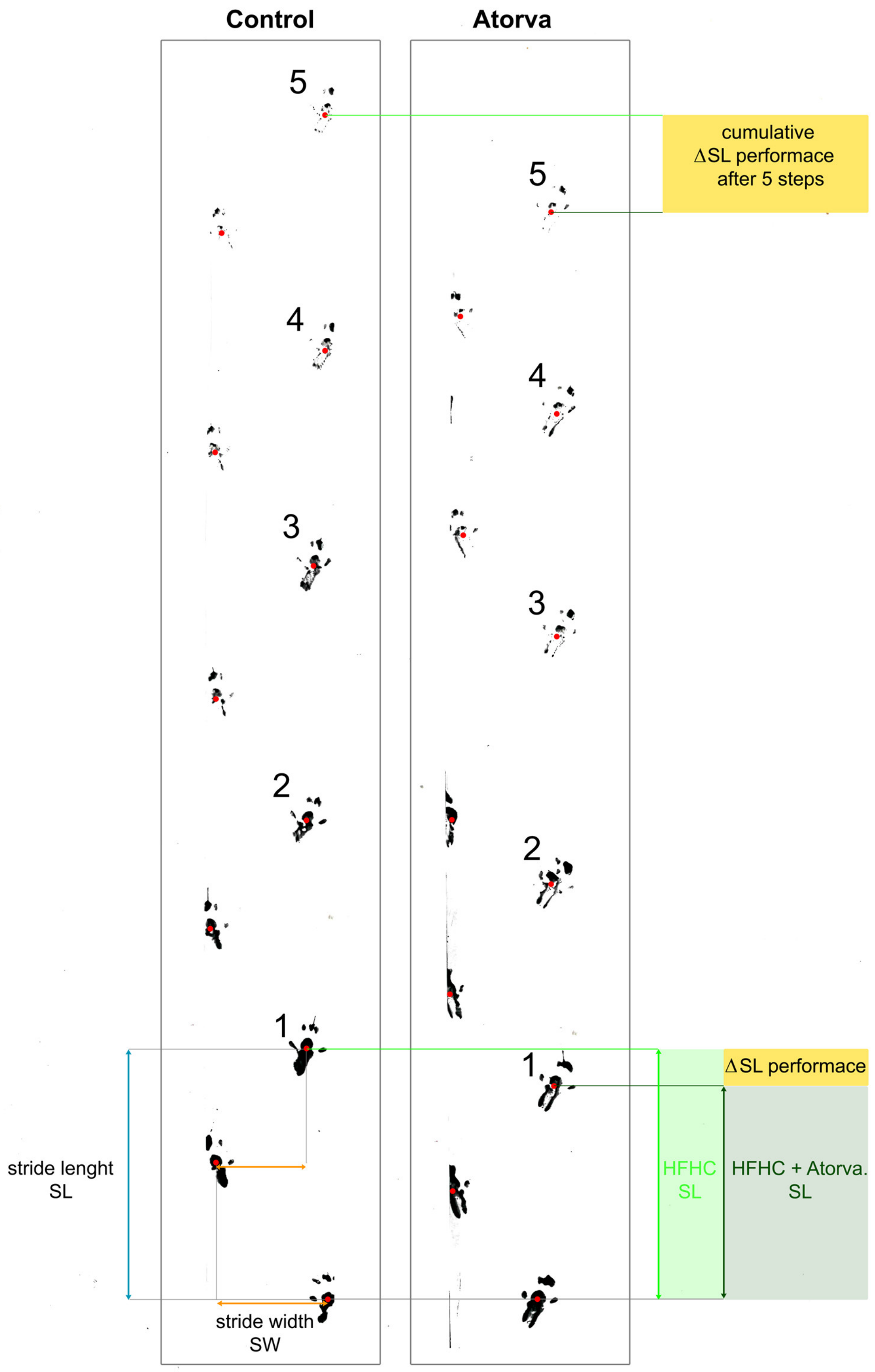

Figure S2. Walking analysis.

Table S1. Diet composition.

D12079B and D19031901

RD Western Diet to Match TD.88137

Formulated by:
Research Diets, Inc.
Sridhar Radhakrishnan
March 2019

Product #	D12079B		D19031901	
			Matching 88137	
%	gm	*kcal*	gm	*kcal*
Protein	20	*17*	20	*17*
Carbohydrate	50	*43*	50	*43*
Fat	21	*40*	21	*40*
Total		*100*		*100*
kcal/gm	4.7		4.7	
Ingredient	gm	*kcal*	gm	*kcal*
Casein	195	*780*	195	*780*
DL-Methionine	3	*12*	3	*12*
Corn Starch	50	*200*	150	*600*
Maltodextrin 10	100	*400*	0	*0*
Sucrose	341	*1364*	341.46	*1365.84*
Cellulose, BW200	50	*0*	50	*0*
Milk Fat, Anhydrous	200	*1800*	210	*1890*
Corn Oil	10	*90*	0	*0*
Ethoxyquin	0.04	*0*	0.04	*0*
Mineral Mix S10001	35	*0*	35	*0*
Calcium Carbonate	4	*0*	4	*0*
Vitamin Mix V10001	10	*40*	10	*40*
Choline Bitartrate	2	*0*	2	*0*
Cholesterol	1.5	*0*	1.5	*0*
GSKG	0	*0*	0	*0*
FD&C Yellow Dye #5	0	*0*	0	*0*
FD&C Red Dye #40	0	*0*	0.05	*0*
FD&C Blue Dye #41	0	*0*	0	*0*
Total	1001.54	*4686*	1002.05	*4688*

Research Diets, Inc.
20 Jules Lane
New Brunswick, NJ 08901 USA
info@researchdiets.com

D19031901.for

www.ResearchDiets.com
